# Beyond the General Linear Model: Linear Mixed-Effects Modeling of fMRI Data from the Human Connectome Project

**DOI:** 10.1007/s12021-026-09820-2

**Published:** 2026-09-29

**Authors:** Daniele Orzechowski, Ronaldo Martins da Costa

**Affiliations:** 1 Department of Informatics and Statistics, Federal University of Santa Catarina, Florianópolis, SC 88040-900 Brazil; 2 Institute of Informatics, Federal University of Goiás, Goiânia, GO 74690-900 Brazil

**Keywords:** fMRI, Linear mixed-effects models, Human connectome project, Reproducible pipelines, Intraclass correlation, Implementation validation

## Abstract

We present a validated, containerised pipeline for Linear Mixed-Effects (LME) analysis of task fMRI, built for reproducibility: the fitting backend must be declared explicitly, outputs are versioned, and every step records a manifest of software, hardware, and parameters. The implementation is cross-validated against the R reference standard lme4 (mean $\boldsymbol{|\Delta \text {ICC}| = 2.2\times 10^{-6}}$ across 21 regions; significance concordance 21/21) and benchmarked at $\boldsymbol{19\times }$ the per-voxel cost of the GLM, completing a whole-brain analysis in under six hours with 100% convergence. In a case study on emotion task data from 142 HCP subjects (283 runs), voxelwise agreement with the conventional General Linear Model (GLM) is high ($\boldsymbol{r = 0.94}$), with LME more conservative under FDR correction (74,004 vs. 108,627 significant voxels at $\boldsymbol{q < 0.05}$; Dice $\boldsymbol{= 0.77}$). An intermediate LME omitting the run fixed effect is almost indistinguishable from the GLM (Dice $\boldsymbol{= 0.99}$), locating the sensitivity difference in the run covariate rather than in hierarchical variance decomposition. Whole-brain ICC mapping shows heterogeneous within-subject variability (mean ICC $\boldsymbol{= 0.199}$), and run effects are significant in 13/21 ROIs, surviving adjustment for head motion. This decomposition strategy offers a reusable diagnostic for sensitivity differences between statistical approaches.

**Supplementary Information:**

The online version contains supplementary material available at https://doi.org/10.1007/s12021-026-09820-2.

## Introduction

The General Linear Model (GLM) has been the cornerstone of fMRI analysis for over two decades (Friston et al., 1994). In the standard two-level approach, first-level models estimate activation parameters per run, and second-level models treat subject-averaged estimates as independent observations for population inference. This framework underpins most neuroimaging software (Cox, 1996; Friston et al., 1994; Woolrich et al., 2004).

However, the GLM has important limitations for hierarchical fMRI data. It treats repeated measurements as independent, potentially inflating false positive rates (Mumford & Nichols, 2006). The summary-statistics approach is asymptotically equivalent to a full mixed-effects model only when first-level variance estimates are homogeneous (Mumford & Nichols, 2006). Additionally, averaging across runs discards within-subject variability that may contain meaningful information about neural processes or individual differences (Garrett et al., 2013; Noble et al., 2019).

Linear Mixed-Effects (LME) models address these issues by explicitly modeling both fixed effects and random effects (Bates et al., 2015), offering proper correlation modeling, variance component estimation via the intraclass correlation coefficient (ICC), and incorporation of run-level covariates. Several studies have explored LME for fMRI: Beckmann et al. (2003) demonstrated improved sensitivity with hierarchical models in FSL; Mumford and Nichols (2006) characterized when summary statistics are suboptimal; Chen et al. (2013) developed 3dLME in AFNI. More recently, reproducibility studies have highlighted large individual differences in task-evoked reliability (Caceres et al., 2009; Elliott et al., 2020; Noble et al., 2019; Zuo & Xing, 2014).

Despite these important contributions, LME adoption in routine fMRI practice remains limited, and the obstacles are as much practical as statistical. Robust implementations exist in R, but Python-based neuroimaging workflows lack a validated, benchmarked LME component that can be dropped into an existing pipeline; the computational cost, convergence behaviour, and implementation fidelity of such a component have not been systematically documented. Two substantive questions are also unresolved: existing comparisons have not isolated the *sources* of divergence between GLM and LME, whether sensitivity differences arise from hierarchical variance decomposition, from run-level covariates, or from both, and whole-brain voxelwise ICC mapping has rarely been performed at the scale afforded by datasets such as the Human Connectome Project (HCP).

The primary contribution of this paper is a tool. We describe and release a validated, benchmarked, containerised LME workflow for volumetric task fMRI, and then use it as a case study to address the two substantive questions above. Our contributions are:

1. **A validated LME pipeline for task fMRI.** We provide an open-source (MIT), Dockerised Python workflow built on statsmodels, cross-validated against the R reference implementation lme4 across all 21 ROIs (mean $|\Delta \text {ICC}| = 2.2\times 10^{-6}$; significance concordance 21/21) and verified by parameter recovery simulations across five variance regimes. The pipeline requires its fitting backend to be declared explicitly and refuses to substitute one engine for another silently, writes versioned outputs so repeated executions cannot overwrite one another, and emits a machine-readable manifest of backend, package versions, hardware, and parameters for every analysis step (Sect. “Reproducibility Infrastructure”).
2. **Computational characterization for whole-brain LME.** We report wall-clock time, memory, and convergence across subject counts (20–200) and voxel counts (100–10,000), together with observed whole-brain performance (215,265 voxels in 5 h 41 min on twelve cores, 100% convergence), giving concrete planning figures for researchers considering voxelwise LME.
3. **A decomposition strategy for diagnosing sensitivity differences.** By fitting an intermediate LME that retains the hierarchical structure but omits the run fixed effect, the pipeline separates the contribution of variance decomposition from that of run-level covariates. Applied here, this shows that hierarchical modelling alone is almost indistinguishable from summary statistics (*r = 0.994*; Dice *= 0.993*) and locates the entire sensitivity difference in the run covariate.
4. **Whole-brain ICC maps as a reusable data product.** We provide dense voxelwise ICC maps (215,265 voxels) revealing a subcortical–cortical gradient in within-subject reliability, extending ROI-level reliability findings (Caceres et al., 2009; Elliott et al., 2020; Noble et al., 2019; Zuo & Xing, 2014) to the whole brain volume, and released unthresholded for reuse.

In the case study we expected the GLM to identify more significant voxels, LME to reveal substantial within-subject variability, both methods to show high stability, and LME to require substantially more computation.

## Methods

### Dataset

We analyzed data from 143 unrelated healthy adults (aged 22–35) from the HCP S1200 release (Van Essen et al., 2013) (Table 1). All participants provided informed consent under protocols approved by Washington University in St. Louis. Framewise displacement (FD) was computed per run following Power et al. (2012), converting the rotational parameters—stored in degrees in the HCP movement regressor files—to millimetres of displacement on a 50 mm sphere. Across the 286 runs, mean FD was *0.1604 ± 0.0606* mm (range 0.0717–0.5141 mm). Runs were excluded if >20% of frames exceeded FD *> 0.5* mm; three runs met this criterion, belonging to two subjects (one in both runs, one in a single run). The analysed sample is therefore 142 subjects contributing 283 runs.

**Table 1:** Sample characteristics

Characteristic	Value
*N* (subjects assessed)	143
*N* (subjects analysed)	142
Age range	22–35 years
Runs per subject	2 (LR + RL)
Total runs acquired	286
Runs excluded (motion)	3
Total runs analysed	283
Mean FD	*0.1604 ± 0.0606* mm

Functional images were acquired on a Siemens Skyra 3T scanner (TR *= 0.72* s; TE *= 33.1* ms; flip angle $= 52^\circ$; 2.0 mm isotropic; 72 slices; multiband factor *= 8*; 176 volumes/run) (Glasser et al., 2013). The emotion task contrasts fearful face processing with shape matching in a blocked design (three emotion, three shape blocks per run; six trials/block at 2 s each) (Barch et al., 2013). Two runs with opposite phase-encoding directions (LR, RL) yielded 286 total runs.

Data underwent HCP minimal preprocessing (Glasser et al., 2013), including distortion correction, motion correction, MNI alignment, intensity normalization, and ICA-FIX artifact removal. All analyses were performed on the volumetric data in MNI152NLin6Asym space at 2 mm isotropic resolution; “voxels” refers to volumetric elements throughout. Identical 5 mm FWHM spatial smoothing was applied to all beta maps before group-level analysis.

### Statistical Analyses

#### First-Level GLM

For each run, a GLM was estimated with regressors for fearful faces and neutral shapes (convolved with canonical HRF), 12 motion regressors (three translations, three rotations, and their temporal derivatives), and cosine high-pass filtering (128 s cutoff), with AR(1) noise modeling. The resulting design matrix contains 16 columns; the pipeline verifies after fitting that all 12 motion regressors are present and aborts otherwise (Sect. “Reproducibility Infrastructure”). The contrast of interest was [fear > neut], yielding 283 beta maps.

#### Second-Level GLM

Beta maps were averaged across retained runs per subject, then entered into a one-sample *t*-test (*n = 142*). Maps were thresholded at FDR *q < 0.05* (primary) and *Z > 3.1* uncorrected (secondary). The second-level GLM was restricted to the same group mask as the LME (Sect. “Voxelwise Analysis”), so that both methods are evaluated over an identical voxel set with the same Benjamini–Hochberg denominator.

#### Linear Mixed-Effects Model

All 283 run-level beta maps were entered into an LME model:

1 $$\begin{aligned} \beta _{ij} = \gamma _0 + \gamma _1 \cdot \text {run}_{ij} + u_{0i} + \epsilon _{ij} \end{aligned}$$

where $\gamma _0$ is the fixed intercept, $\gamma _1$ the fixed run effect (LR vs. RL), $u_{0i} \sim N(0, \tau ^2)$ the random intercept, and $\epsilon _{ij} \sim N(0, \sigma ^2)$ the residual. The ICC quantifies between-subject variance proportion: $\text {ICC} = \tau ^2/(\tau ^2 + \sigma ^2)$. Statistical inference used Wald *z*-tests, appropriate given *n = 142* (Luke, 2017). Models were fit via REML using Powell optimization (statsmodels v0.14.6). Convergence is reported from the optimizer’s own flag rather than from the absence of an exception.

An intermediate model omitting the run covariate ($\beta _{ij} = \gamma _0 + u_{0i} + \epsilon _{ij}$) was fit to isolate hierarchical variance decomposition from run effects. A random-intercept-only specification was used because with two runs per subject, random-slope models are nearly saturated; simulations confirmed that random-intercept was preferred by AIC in 95.4% of runs (see Sect. “Simulation Analyses”).

#### Equalized Threshold and Cluster-Level Correction

FDR *q < 0.05* was applied independently to each method (primary comparison). Additionally, cluster-level FWE correction (*Z > 3.1* cluster-forming threshold) was applied to the GLM *z*-map (Table 5).

### Implementation Validation

Three validation analyses establish that the statsmodels backend reproduces the reference implementation. (1) **Parameter recovery**: 100 Monte Carlo simulations per scenario across five variance regimes showed small biases (intercept bias: $|{<}0.03|$; ICC bias: $|{<}0.04|$; Table 2). (2) **Cross-validation against R**lme4 : the identical ROI dataset was fitted under both backends in separate, independently archived executions—statsmodels v0.14.6 and lme4 v2.0.6 via pymer4 v0.9.2—and the two output files were compared directly. Agreement across all 21 ROIs was mean $|\Delta \text {ICC}| = 2.2\times 10^{-6}$ (maximum $1.6\times 10^{-5}$), with significance concordance in 21 of 21 regions (Table 3). (3) **ROI-level consistency**: LME intercepts agreed with *t*-test means (Table 4).

The two backends differ in one respect that is inferential rather than estimative: lmerTest applies Satterthwaite degrees of freedom whereas statsmodels uses Wald *z*-tests. This places one region (right hippocampus, *p = 0.056* vs. *p = 0.058*) on either side of the 0.05 threshold while the underlying estimates agree to within $10^{-6}$. Researchers comparing results across backends should expect this class of discrepancy at threshold boundaries.

**Table 2:** Parameter recovery: mean estimates (± SD) across 100 simulations per scenario

Scenario	True $\gamma _0$	Est. $\gamma _0$	True ICC	Est. ICC
Moderate effect	0.300	*0.282 ± 0.087*	0.200	*0.208 ± 0.083*
Large effect	1.000	*0.986 ± 0.067*	0.123	*0.134 ± 0.080*
Small effect	0.100	*0.078 ± 0.112*	0.308	*0.314 ± 0.078*
High ICC	0.500	*0.490 ± 0.128*	0.900	*0.901 ± 0.015*
Low ICC	0.500	*0.473 ± 0.121*	0.004	*0.043 ± 0.056*

**Table 3:** Cross-validation: statsmodels vs. R lme4 for all 21 ROIs. Both backends were fitted on the identical dataset in separate archived executions

ROI	ICC_sm_	ICC_lme4_	*|Δ *ICC|	Concordant
L Amygdala	0.412419	0.412419	$6.3\times 10^{-7}$	Yes
R Amygdala	0.324947	0.324947	$1.3\times 10^{-8}$	Yes
L Hippocampus	0.193320	0.193317	$3.0\times 10^{-6}$	Yes
R Hippocampus	0.299237	0.299237	$4.1\times 10^{-9}$	Yes
Insular Cortex	0.201175	0.201191	$1.6\times 10^{-5}$	Yes
IFG pars opercularis	0.095089	0.095089	$1.7\times 10^{-8}$	Yes
IFG pars triangularis	0.417096	0.417096	$4.9\times 10^{-7}$	Yes
Anterior Cingulate	0.083210	0.083210	$2.0\times 10^{-8}$	Yes
Paracingulate Gyrus	0.381556	0.381556	$4.3\times 10^{-9}$	Yes
Medial Frontal Cortex	0.432016	0.432016	$3.7\times 10^{-8}$	Yes
Superior Frontal Gyrus	0.142161	0.142146	$1.5\times 10^{-5}$	Yes
Middle Frontal Gyrus	0.133899	0.133899	$3.4\times 10^{-8}$	Yes
Frontal Pole	<0.001	<0.001	$1.1\times 10^{-10}$	Yes
Orbital Frontal Cortex	0.213777	0.213772	$4.5\times 10^{-6}$	Yes
L Thalamus	0.114744	0.114744	$4.5\times 10^{-8}$	Yes
R Thalamus	0.287645	0.287645	$3.2\times 10^{-9}$	Yes
L Caudate	0.059688	0.059687	$3.4\times 10^{-8}$	Yes
L Putamen	0.104388	0.104388	$3.6\times 10^{-8}$	Yes
L Pallidum	0.173836	0.173832	$3.4\times 10^{-6}$	Yes
Brainstem	0.224696	0.224694	$2.6\times 10^{-6}$	Yes
L Cerebral Cortex	0.297968	0.297968	$3.2\times 10^{-8}$	Yes
Mean *|Δ *ICC|			$\mathbf {2.2\times 10^{-6}}$	

ICCs shown to six decimals; all 21 regions agree to at least five. “Concordant” indicates identical significance classification at *q < 0.05*. Both backends converged in all regions

**Table 4:** ROI-level consistency check: LME vs. *t*-test reference

ROI	LME intercept	*t*-test mean	ICC	Converged
Frontal Pole	0.0110	0.0136	<0.001	Yes
Insular Cortex	−0.0102	−0.0145	0.201	Yes
Sup. Frontal Gyrus	−0.0066	0.0071	0.142	Yes
Mid. Frontal Gyrus	0.0555	0.0759	0.134	Yes
IFG pars triangularis	0.0739	0.1038	0.417	Yes

### Reproducibility Infrastructure

Three properties of the released pipeline are design decisions rather than implementation details. Each addresses a failure mode that produces results which cannot be attributed to a stated method, and which typically leaves no trace in the output.

#### Explicit Backend Declaration

The fitting engine must be named by the caller (LME_BACKEND=statsmodels or pymer4); if it is unavailable the pipeline raises an error rather than substituting another, and the resolved engine is written to every output record.

#### Versioned Outputs

Each step takes a run tag appended to all output filenames, so a second execution cannot overwrite the first, and figures are generated from an identically tagged file set.

#### Environment Manifests

Each step writes a JSON manifest recording the resolved backend and package versions, R and lme4 versions where applicable, CPU model, memory, mask voxel count, and all analysis parameters.

Additionally, the first-level model verifies its own design matrix after fitting and aborts if the expected motion regressors are absent, and the group-level scripts log the Benjamini–Hochberg denominator, converting two classes of silent degradation into explicit failures.

### Region-of-Interest Analysis

Twenty-one ROIs were selected from FSL’s Harvard-Oxford atlases (Desikan et al., 2006), comprising cortical emotion and cognitive regions and subcortical structures (see Table 6 for the full list). Masks were thresholded at 25% probability and resampled to the 2 mm analysis grid. Mean beta values were extracted per subject and run (283 observations/ROI).

For GLM, betas were averaged across runs and tested with one-sample *t*-tests. Cohen’s *d* used total variance ($d = \bar{\beta }/\sqrt{\text {Var}(\beta _{ij})}$) for both methods to enable direct comparison; conventional between-subject *d* values are in Supplementary Table S1. LME models used the specification in Eq. 1 with a hierarchical optimization strategy (five algorithms; best log-likelihood retained). FDR correction was applied at *q < 0.05* across 21 ROIs.

### Sensitivity Analyses

To evaluate whether run effects were driven by head motion, we fit an extended LME including mean FD per run as a covariate (Eq. 2) and compared the run coefficient $\gamma _1$ between the two specifications. Note that motion is thereby controlled at two levels: the 12 motion regressors enter the first-level model, and mean FD per run enters the group-level model. Additionally, we computed the autocorrelation function of second-level LME residuals across all 21 ROIs, with Ljung-Box tests (Ljung and Box, 1978) for lag-1 autocorrelation.

2 $$\begin{aligned} \beta _{ij} = \gamma _0 + \gamma _1 \cdot \text {run}_{ij} + \gamma _2 \cdot \text {FD}_{ij} + u_{0i} + \epsilon _{ij} \end{aligned}$$

### Stability Assessment

Effect estimate robustness was evaluated through: (1) **Bootstrap** (1,000 resamples, preserving within-subject structure); (2) **Split-half reliability** (100 random splits; Spearman-Brown corrected: $r_{SB} = 2r_{\text {half}}/(1 + r_{\text {half}})$) (Brown, 1910; Spearman, 1910); and (3) **Leave-one-out CV** (coefficient of variation across iterations).

### Computational Benchmark

Benchmarking used synthetic data (*β = 0.3*, ICC *≈ 0.2*) with systematic variation of subjects (20–200) and voxels (100–10,000). Wall-clock time, memory, and convergence were measured. All analyses reported in this paper were executed on a single cloud instance with 12 Intel Xeon (Cascade Lake) vCPUs and 64 GB RAM under Ubuntu 24.04, with BLAS threading pinned to one thread per worker so that parallelism is controlled solely by the job scheduler.

### Voxelwise Analysis

The group mask was computed from the *unsmoothed* beta maps, retaining voxels with finite non-zero values in *≥ *50% of runs, followed by binary opening, and was then intersected with the standard MNI152 brain mask. This yielded 215,265 voxels, shared by the GLM and both LME models. The mask must be derived before smoothing: applying the same criterion to smoothed maps yields 356,749 voxels, the excess being a halo of non-zero values spread past the cortical boundary by the 5 mm kernel. Since the number of tests is the Benjamini–Hochberg denominator, this affects the significance threshold and every reported percentage.

Both LME models were fit at each voxel, in chunks of 10,000 across 8 parallel workers with checkpointing. Quality control flagged non-converged, non-finite, or extreme values (*|z| > 500*, ICC *∉ [0,1]*), and the number of finite *p*-values entering the FDR step is logged per execution. Variance components are bounded below at zero, so where no between-subject variance is estimable the ICC reaches the boundary and is reported as zero rather than indicating a convergence failure.

### Simulation Analyses

Three simulations were conducted: (1) **False positive rate**: 1,000 null datasets confirmed adequate Type I error (GLM: 0.045 [0.034, 0.060]; LME: 0.052 [0.040, 0.068]). (2) **RI vs. RS model comparison**: random-intercept preferred by AIC in 95.4% of 196 comparisons; 3% RS singularity. (3) **ICC bootstrap stability**: with two runs, ICC SD *= 0.081* (RMSE *= 0.081*), improving to 0.055 with three runs, 0.038 with five, and 0.026 with ten.

### Cluster-Level Statistics

Peak coordinates were extracted from the group GLM *z*-map at *Z > 3.1* using a minimum inter-peak distance of 8 mm. Cluster-level statistics were computed at the same cluster-forming threshold with signed clusters reported separately, since the emotion > shape contrast produces substantial deactivation as well as activation (Table 5). Cluster volumes follow directly from the voxel count at 2 mm isotropic resolution (8 mm$^3$ per voxel). These statistics are derived from the same group mask used for the LME models (Sect. “Voxelwise Analysis”), so cluster extents are directly comparable between methods. These coordinates are reported for reference and for comparison with the published HCP emotion task literature; the equivalent unthresholded maps are released with the pipeline.

**Table 5:** GLM cluster-level statistics (*Z > 3.1* cluster-forming threshold)

Dir.	Size (vox)	Size (mm$^3$)	$Z_\text {peak}$	*x*	*y*	*z*
+	53,497	427,976	15.74	20	−96	−8
−	7,544	60,352	−10.16	−58	−28	42
−	5,518	44,144	−9.76	64	−22	20
−	4,303	34,424	−9.23	10	−30	42
+	1,403	11,224	8.01	−2	32	48
−	1,182	9,456	−6.56	18	−76	−54
−	1,088	8,704	−6.87	−16	−72	−54
−	803	6,424	−6.68	−24	−14	60
+	769	6,152	7.95	−34	−60	46
−	756	6,048	−6.86	−28	40	36

Dir., direction; vox, voxels (2 mm isotropic); MNI coordinates in mm. 159 clusters were identified at *Z > 3.1*

### Statistical Software

Analyses used Python 3.12 with nilearn, statsmodels 0.14.6, scipy, nibabel, and joblib; the cross-backend validation additionally used R 4.3.3 with lme4 2.0.6 and lmerTest, accessed through pymer4 0.9.2. Complete pinned environments for every reported analysis are in the manifests described in Sect. “Reproducibility Infrastructure”. Code: https://github.com/DaniOrze/hcp-emotion-lme (MIT license; Dockerfile included).

## Results

### Group-Level Activation

**Fig. 1:**
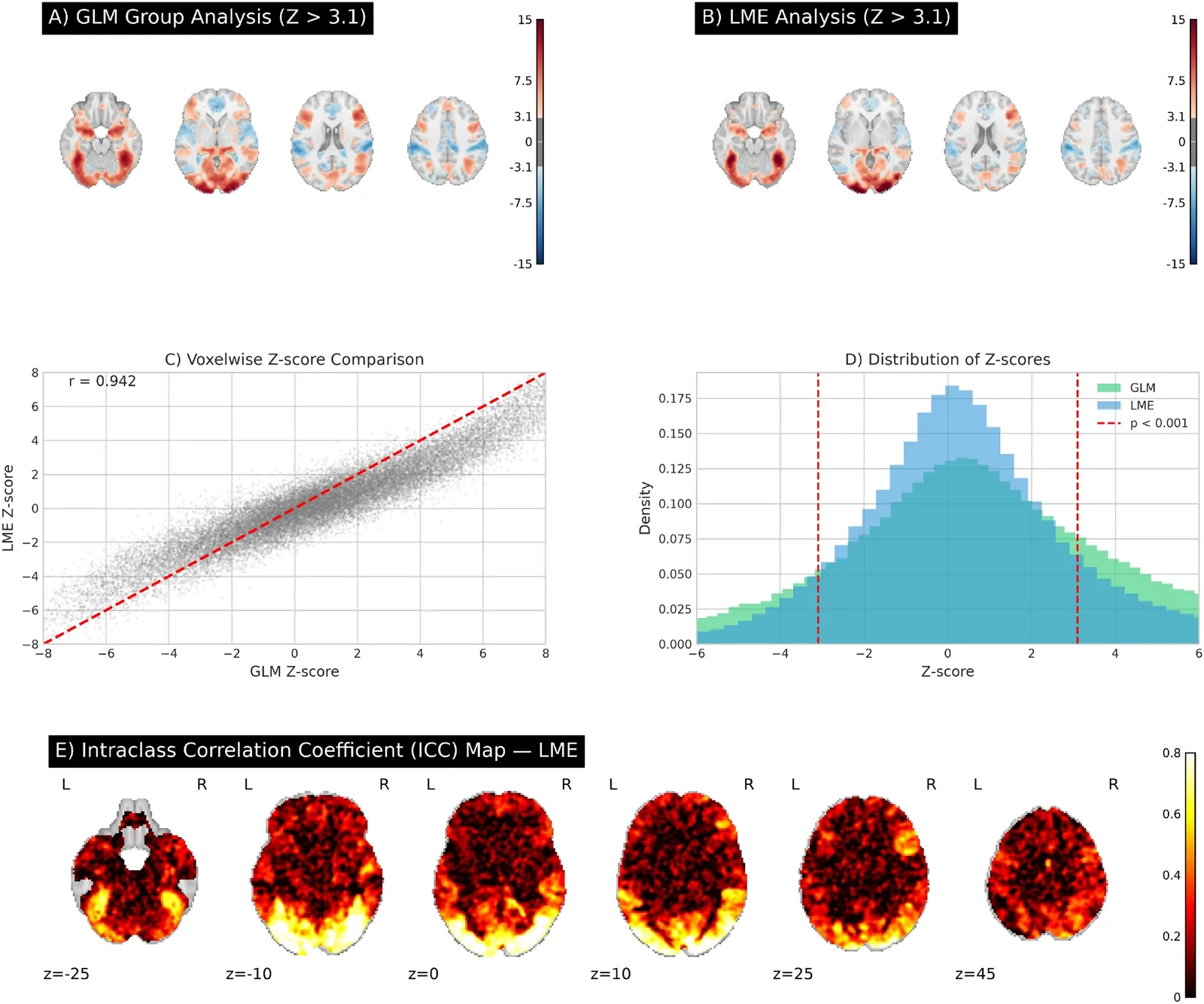
Whole-brain activation and ICC maps for emotion > shape. **(A–B)** Group *z*-maps for the GLM (A) and the full LME (B), thresholded at *Z > 3.1* and displayed on axial slices of the MNI152 template; warm colors denote positive and cool colors negative effects. **(C)** Voxelwise *z*-score scatter across the shared mask of 215,265 voxels (*r = 0.9425*); the dashed line marks identity. **(D)** Distributions of GLM and LME *z*-scores; dashed lines mark *p < 0.001* (two-tailed). **(E)** Voxelwise ICC map from the LME, on axial slices at *z = -25, -10, 0, 10, 25, 45* mm; warm colors = higher within-subject reliability

Both methods identified a highly similar network including bilateral amygdala, anterior insula, inferior frontal gyrus, dorsomedial prefrontal cortex, and visual cortex (Fig. 1). Over the shared mask of 215,265 voxels at FDR *q < 0.05*, the GLM identified 108,627 significant voxels against 74,004 for the full LME (ratio 1.47*× *; Dice *= 0.769*); at *Z > 3.1* the ratio was 1.58*× *. Voxelwise *z*-score correlation was *r = 0.9425*, and Dice overlap declined only gradually with threshold (0.804 at *Z > 2.0*; 0.733 at *Z > 5.0*), indicating that the two maps differ in extent rather than in spatial configuration.

The intermediate LME, which retains the hierarchical structure and differs from the full model only in omitting the run covariate, identified 109,273 significant voxels and was almost indistinguishable from the GLM (*r = 0.9942*; Dice *= 0.993*). The sensitivity difference is therefore attributable to that covariate rather than to hierarchical variance decomposition.

The ICC map revealed substantial spatial heterogeneity (mean *= 0.199*, median *= 0.150*, range 0.000–0.912; Fig. 1E), with higher values in subcortical structures and primary sensory cortex and lower values in prefrontal regions. All 215,265 voxels converged.

### ROI Comparison

**Fig. 2:**
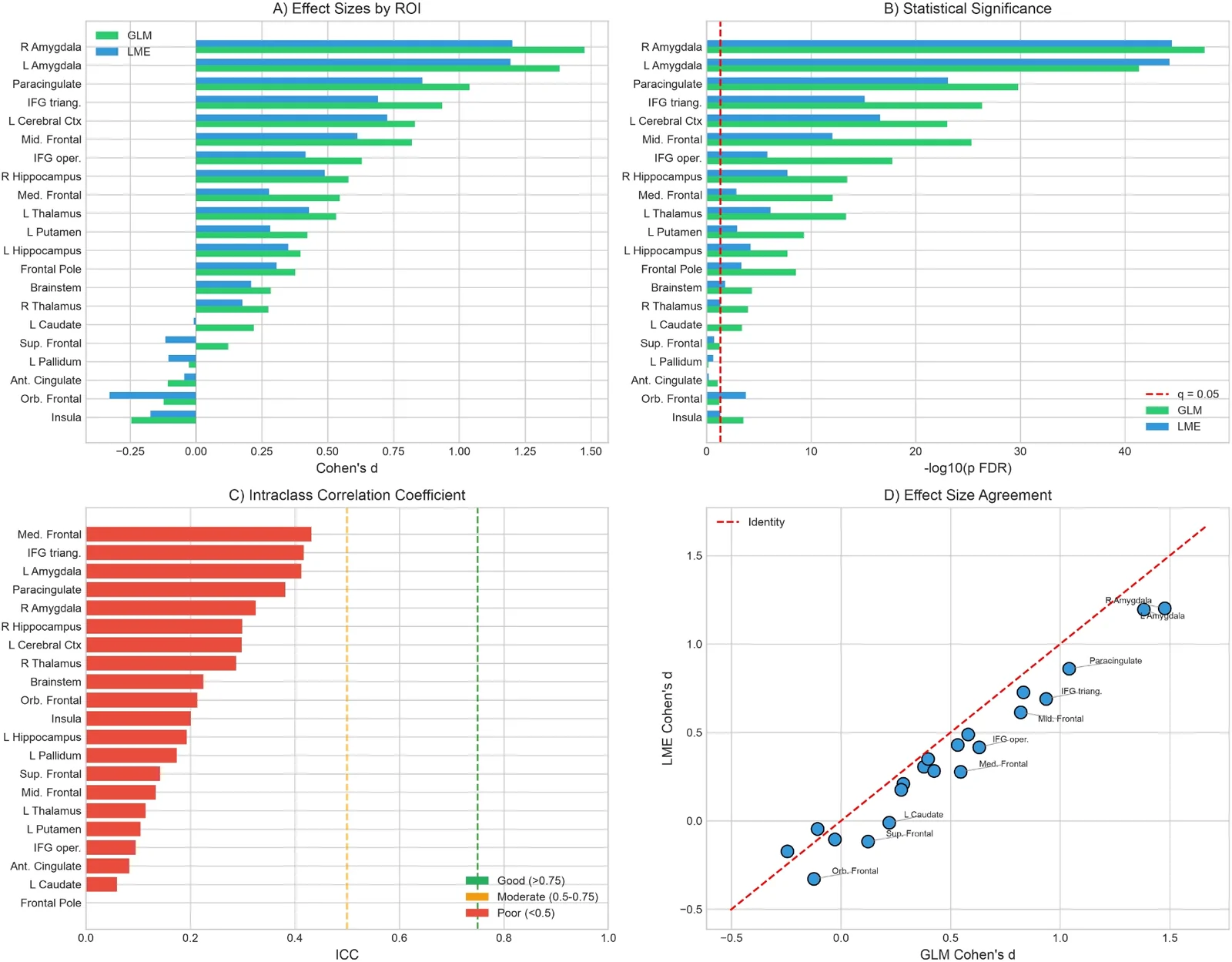
ROI comparison across the 21 ROIs. **(A)** Cohen’s *d* per ROI for the GLM (green) and the LME (blue), ordered by GLM effect size. **(B)** Significance for both methods, as $-\log _{10}(p_{\text {FDR}})$; the dashed line marks *q = 0.05*. ROI order as in (A). **(C)** LME intraclass correlation per ROI, ordered by ICC and coloured by conventional reliability bands (dashed lines at 0.5 and 0.75); all ROIs fall in the poor range, and the frontal pole is at the boundary value of zero (see text). Bootstrap 95% CIs are given in Table 6. **(D)** LME versus GLM Cohen’s *d*; the dashed line marks identity, and points below it indicate the more conservative LME estimates

For the task contrast, 17 of 21 ROIs were significant by GLM and 17 by LME (FDR *q < 0.05*), with 16 detected by both (Table 6; Fig. 2A–B). Mean absolute Cohen’s *d* was 0.542 (GLM) and 0.429 (LME), a 21% reduction, with the two methods highly concordant across regions (Fig. 2D). Mean ICC across the 21 ROIs was 0.219, compared with 0.199 across the 215,265 voxels of the whole-brain map (Sect. “Group-Level Activation”).

**Table 6:** ROI analysis comparing GLM and LME (*n = 142* subjects, 283 runs). ICC bootstrap 95% CIs from 2,000 iterations. Cohen’s *d* uses total variance for both methods; conventional *d* in Supplementary Table S1

	GLM		LME		
ROI	*β * (SE)	*t*	*β * (SE)	*z*	ICC [95% CI]
L Amygdala	0.237 (0.012)	20.05***	0.200 (0.014)	14.24***	0.412 [0.218, 0.570]
R Amygdala	0.239 (0.010)	23.04***	0.182 (0.013)	14.33***	0.325 [0.162, 0.468]
L Hippocampus	0.045 (0.007)	6.07***	0.040 (0.010)	4.18***	0.193 [0.015, 0.348]
R Hippocampus	0.062 (0.007)	8.60***	0.052 (0.009)	5.82***	0.299 [0.145, 0.433]
Insular Ctx	−0.015 (0.004)	−3.78***	−0.010 (0.005)	−2.06*	0.201 [0.044, 0.360]
IFG pars oper.	0.071 (0.007)	10.33***	0.046 (0.009)	4.96***	0.095 [0.000, 0.263]
IFG pars triang.	0.104 (0.008)	13.74***	0.074 (0.009)	8.23***	0.417 [0.246, 0.566]
Anterior Cing.	−0.008 (0.004)	−1.74	−0.003 (0.006)	−0.53	0.083 [0.000, 0.320]
Paracingulate G.	0.146 (0.010)	15.14***	0.119 (0.012)	10.25***	0.382 [0.235, 0.516]
Medial Frontal Ctx	0.057 (0.007)	8.00***	0.028 (0.008)	3.31**	0.432 [0.292, 0.556]
Sup. Frontal G.	0.007 (0.004)	1.99	−0.007 (0.005)	−1.38	0.142 [0.003, 0.287]
Mid. Frontal G.	0.076 (0.006)	13.30***	0.056 (0.008)	7.31***	0.134 [0.000, 0.302]
Frontal Pole	0.014 (0.002)	6.45***	0.011 (0.003)	3.65***	<0.001 [0.000, 0.178]
Orbital Frontal Ctx	−0.014 (0.007)	−1.92	−0.036 (0.009)	−3.91***	0.214 [0.097, 0.328]
L Thalamus	0.048 (0.006)	8.53***	0.039 (0.008)	5.13***	0.115 [0.000, 0.320]
R Thalamus	0.020 (0.005)	4.05***	0.013 (0.006)	2.11*	0.288 [0.156, 0.403]
L Caudate	0.033 (0.009)	3.68***	−0.001 (0.012)	−0.12	0.060 [0.000, 0.226]
L Putamen	0.049 (0.007)	6.82***	0.032 (0.010)	3.36**	0.104 [0.000, 0.271]
L Pallidum	−0.004 (0.010)	−0.44	−0.016 (0.013)	−1.25	0.174 [0.010, 0.338]
Brainstem	0.013 (0.003)	4.32***	0.009 (0.004)	2.50*	0.225 [0.050, 0.379]
L Cerebral Ctx	0.021 (0.002)	12.37***	0.019 (0.002)	8.66***	0.298 [0.108, 0.463]

*β * = mean beta (BOLD units); SE = standard error; L = Left; R = Right; Ctx = Cortex; G. = Gyrus; Cing. = Cingulate; oper. = opercularis; triang. = triangularis; Sup. = Superior; Mid. = Middle; IFG = inferior frontal gyrus; ICC = intraclass correlation (LME). Asterisks denote FDR-corrected significance: *q < 0.05, **q < 0.01, ***q < 0.001

### Within-Subject Variability and Run Effects

ICC ranged from below 0.001 (frontal pole) to 0.412 (left amygdala), with subcortical regions showing higher values (amygdala mean *= 0.37*) than lateral frontal cortex (${\sim }0.10$–0.14). The wide bootstrap CIs, especially for low-ICC ROIs where the lower bound reaches zero, reflect the imprecision of two-run ICC estimation (simulation RMSE *= 0.081*). In the frontal pole the between-subject variance reached the lower boundary, so the ICC is reported as effectively zero; its bootstrap interval, [0.000, 0.178], indicates that no between-subject variance is estimable with two runs rather than that the model failed.

Turning from the task contrast to the run covariate, run effects were significant in 13 of the 21 ROIs (mean absolute effect *= 0.0358*; maximum *= 0.1147* in right amygdala). Adding mean FD per run to the group-level model left them essentially unchanged, where all 13 remained significant, the mean absolute effect moved to 0.0356, and the FD coefficient was itself significant in only 1 of 21 ROIs. With 12 motion regressors already in the first-level models, run effects are thus controlled for motion at both levels and survive, supporting phase-encoding-related rather than motion-driven origins, see Sect. “Limitations” regarding acquisition order.

### Residual Autocorrelation

**Fig. 3:**
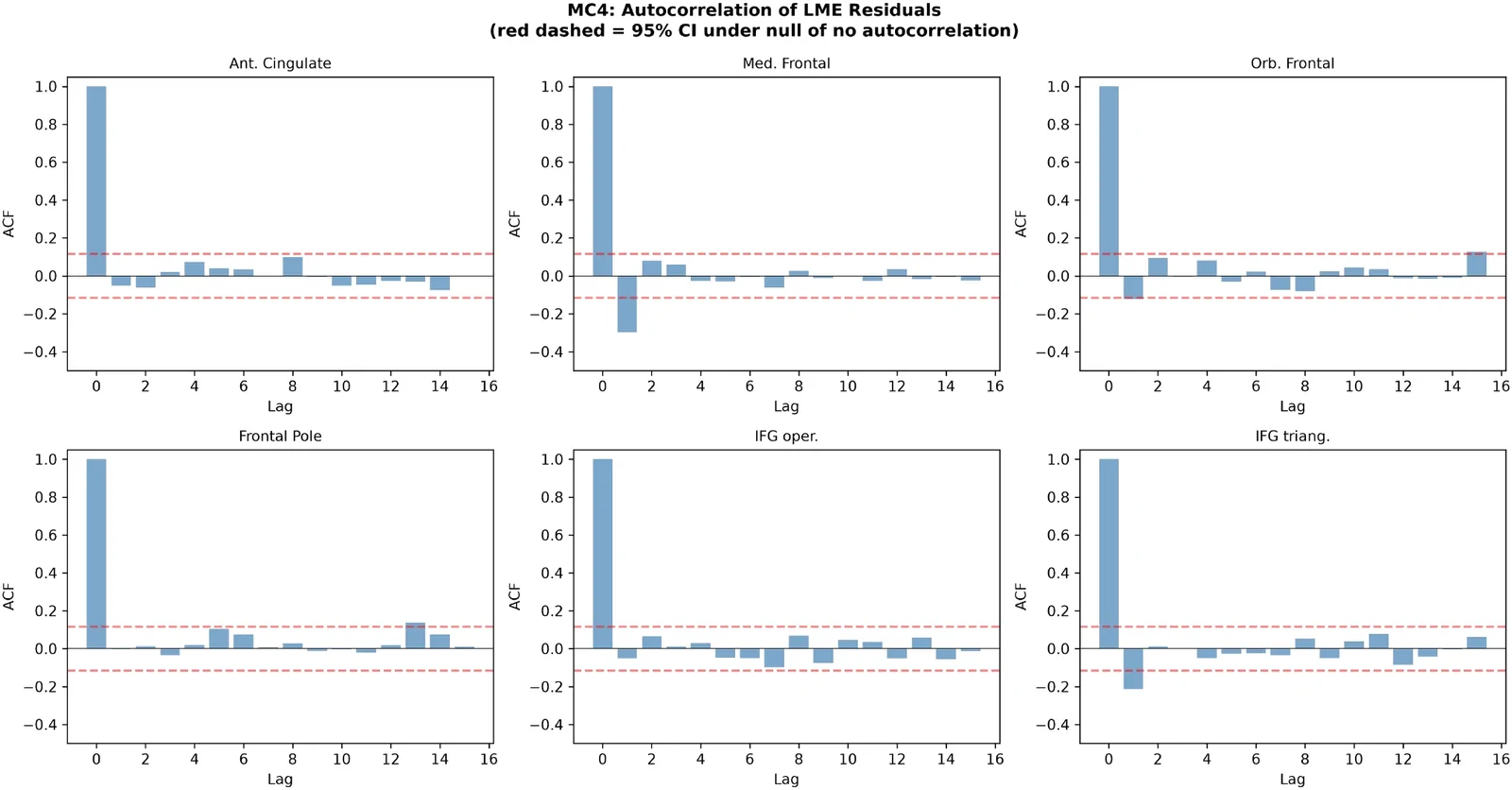
Residual autocorrelation of the second-level LME. Autocorrelation function for six representative ROIs; the red dashed lines mark the 95% confidence band under the null of no autocorrelation. Lag-1 autocorrelation was negative in 20 of 21 ROIs and significant by Ljung-Box test in 13, consistent with the LR–RL alternation design

Lag-1 autocorrelation was negative in 20 of 21 ROIs (mean *= -0.138*), reaching significance in 13 by Ljung-Box test; the single exception (left putamen, ACF*(1) = 0.007*) was indistinguishable from zero. This reflects the mechanical structure of the random-intercept model rather than misspecification (Fig. 3).

### Stability

**Fig. 4:**
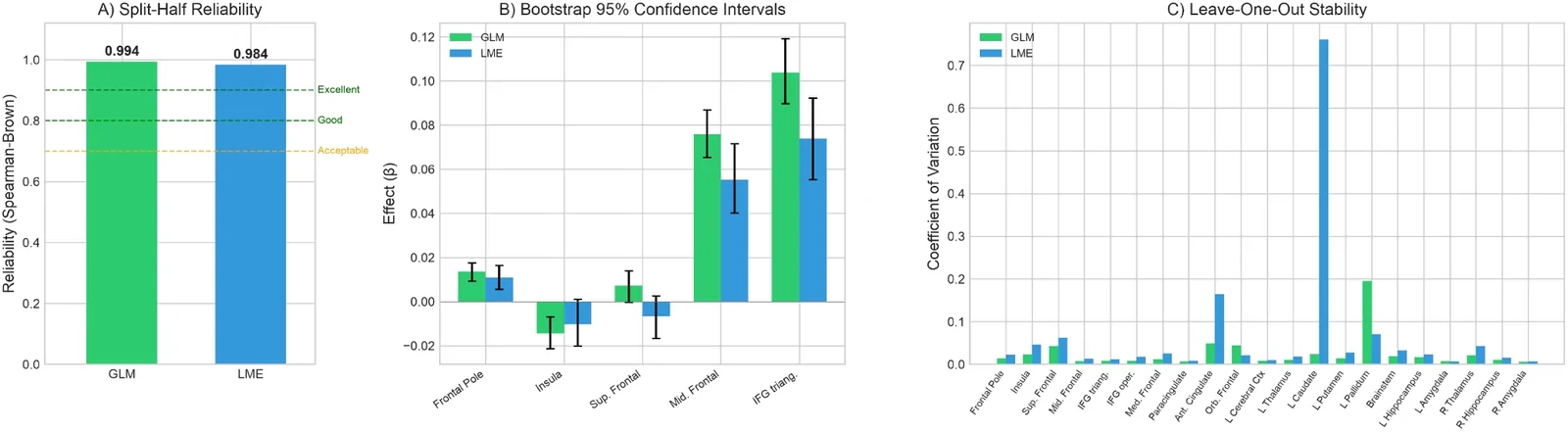
Stability analysis, GLM (green) versus LME (blue). **(A)** Split-half reliability over 100 random splits, Spearman-Brown corrected; dashed lines mark conventional acceptable, good, and excellent thresholds. **(B)** Group effect estimates (*β *) with bootstrap 95% CIs (2,000 resamples) for five representative ROIs. **(C)** Coefficient of variation of the group effect across leave-one-out iterations, per ROI

Split-half reliability was excellent (GLM: $r_{SB} = 0.994$; LME: $r_{SB} = 0.984$). Leave-one-out CV was low on average (GLM: 0.026; LME: 0.067), confirming robustness to individual subjects (Fig. 4). The single elevated value (left caudate, CV *= 0.76*) reflects that region’s near-zero effect estimate (*β = -0.001*, $p_{\text {FDR}} = 0.907$) inflating a ratio-based measure, rather than genuine instability.

### Computational Efficiency

**Fig. 5:**
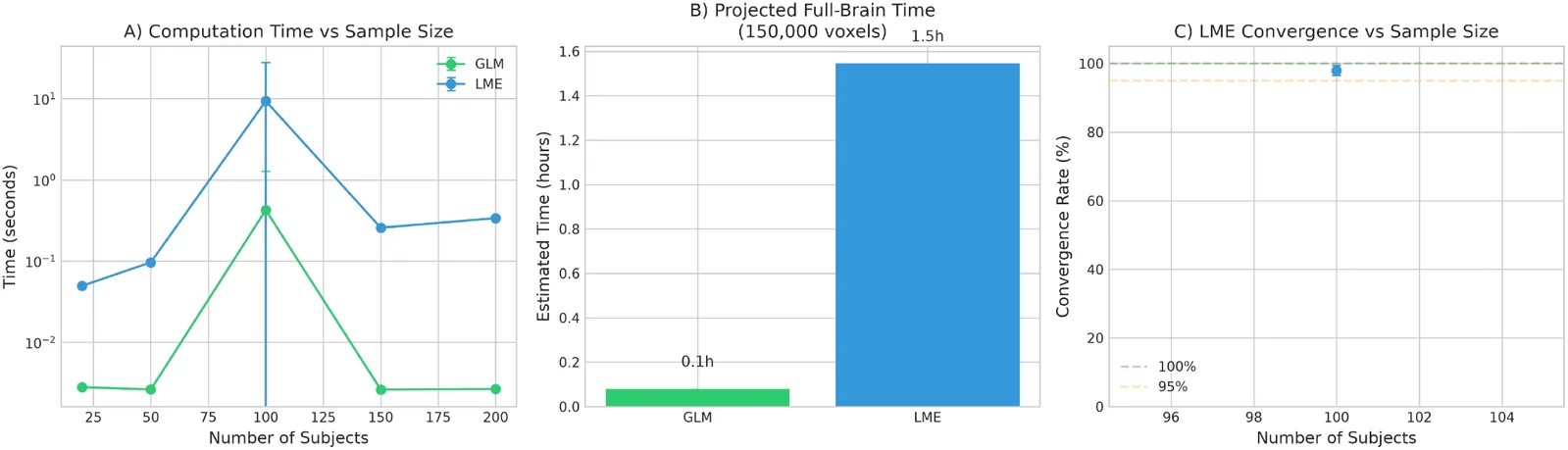
Computational benchmark. **(A)** Time vs. number of subjects. **(B)** Projected full-brain time: GLM *= 0.08* h, LME *= 1.55* h (observed: 5 h 41 min for 215k voxels on 8 workers). **(C)** LME convergence rate across voxel counts (96–100% at *n = 100* subjects)

Mean time per voxel in the synthetic benchmark was 1.99 ms (GLM) and 37.20 ms (LME), a factor of 19. Extrapolated single-threaded times for 150,000 voxels: 0.08 h (GLM) vs. 1.55 h (LME). The observed whole-brain analysis (215,265 voxels, 8 parallel workers) required 5 h 41 min for the full model and 5 h 39 min for the intermediate model; the gap relative to the extrapolation reflects checkpoint I/O, convergence heterogeneity, and memory pressure not captured by synthetic data. Convergence, read from the optimizer flag, was 100% on the real data and averaged 98% in the synthetic benchmark (Fig. 5).

### Voxelwise LME Summary

Full model: 74,004 significant voxels (34.4% of 215,265); mean ICC *= 0.199* (median 0.150); convergence 100%. Intermediate model: 109,273 significant (50.8%); mean ICC *= 0.194* (median 0.146); convergence 100%. The Benjamini–Hochberg denominator was 215,265 in both cases, with no fits discarded. Subcortical structures showed higher ICCs than association cortices.

## Discussion

### Main Findings

The primary contribution of this work is a validated and benchmarked LME pipeline for volumetric task fMRI, together with the reproducibility properties described in Sect. “Reproducibility Infrastructure”. The case study demonstrates what the tool makes visible. By introducing an intermediate LME model that retains the hierarchical structure but omits the run covariate, we show that the commonly observed sensitivity difference between these frameworks is not an inherent consequence of mixed-effects modeling, but rather reflects the absorption of systematic run-related variance. This reframes the GLM–LME comparison: when no run-level covariates are modeled, the two approaches yield nearly identical results (*r = 0.9942*; Dice *= 0.993*), suggesting that previous reports of reduced LME sensitivity may have conflated two distinct effects. This decomposition is not specific to run effects, it generalises to any run-level covariate, and we suggest it as a routine diagnostic when comparing statistical frameworks. Additionally, the whole-brain ICC maps reveal a pronounced subcortical–cortical gradient in within-subject reliability, with implications for the design of brain–behavior correlation studies and biomarker development.

The complete set of case-study findings is: (1) under equalized FDR correction over a shared mask, the full LME identifies *1.47× * fewer significant voxels, but an intermediate model without the run effect is almost indistinguishable from the GLM (Dice *= 0.993*), attributing the difference to the run covariate; (2) ROI effect sizes are concordant, with LME consistently lower (mean |*d*| 0.542 vs. 0.429); (3) within-subject variability is substantial (mean voxelwise ICC *= 0.199*) and spatially heterogeneous; (4) run effects are significant in 13/21 ROIs and all 13 survive adjustment for framewise displacement, with motion controlled at both analysis levels; (5) both methods show excellent stability ($r_{\text {SB}} > 0.98$); (6) LME is *19× * slower per voxel but achieves 100% convergence and completes a whole-brain analysis in under six hours on twelve cores; and (7) the statsmodels implementation agrees with lme4 to the sixth decimal place.

### The Role of the Run Covariate

A central insight is that the GLM-LME sensitivity difference is driven by the run fixed effect, not by hierarchical variance decomposition. The intermediate LME produced near-identical results to GLM (*r = 0.9942*), while the full LME with run effect showed moderate divergence (*r = 0.9425*). The run covariate absorbs systematic phase-encoding-related variance, reducing apparent activation extent. Researchers should consider whether run effects represent nuisance variance to control or are incidental to the research question. The pipeline supports this decision directly: fitting both specifications and comparing them is a single additional command, and the resulting difference quantifies how much of the apparent activation depends on run-level structure.

### When to Use LME vs. GLM

GLM is preferable for single-run designs or rapid exploratory analysis. Our intermediate model analysis further suggests that when run-level covariates are not of interest, the GLM remains a practical and nearly equivalent choice, as hierarchical variance decomposition alone produces negligible divergence from standard summary statistics (*r = 0.9942*). LME is preferable when multiple runs are available and within-subject variability is of interest, when run-level covariates need explicit modeling, when conservative inference is prioritized, or when ICC-based reliability is relevant to research questions.

### ICC Interpretation

The ICCs reported here are specific to the emotion > shape contrast in 142 healthy young adults (Bennett and Miller, 2010). High-ICC regions (e.g., amygdala, ${\sim }0.32$–0.41) may be suitable for brain-behavior correlations (Elliott et al., 2020; Noble et al., 2019); low-ICC regions show high within-subject variability. However, the within-subject variance ($\sigma ^2$) conflates genuine neural variability (Garrett et al., 2013), measurement noise, and model misspecification. Furthermore, two-run ICC estimates carry substantial uncertainty (RMSE *= 0.081*); precision improves substantially with three (SD *= 0.055*) or more runs.

### Methodological Considerations

The statsmodels implementation agreed with R lme4 to mean $|\Delta \text {ICC}| = 2.2\times 10^{-6}$, with the only practical divergence arising from the differing degrees-of-freedom approximations at threshold boundaries. Residual autocorrelation (mean lag-1 *= -0.138*) reflects the run-alternation design rather than misspecification. Convergence was 100% when read from the optimizer’s own flag; we note that a convergence rate computed from the absence of exceptions rather than from the optimizer state will overstate model fit, and recommend that pipelines report the latter.

### Generalizability and Extensibility

Since the pipeline is the primary contribution, its scope of application deserves explicit statement.

#### Other Tasks and Atlases

The workflow applies without modification to any task for which run-level contrast maps can be produced; the task name, condition labels, and contrast definition are configuration values rather than code. Likewise, the ROI module accepts any probabilistic or labelled atlas that can be resampled to the analysis grid, the Harvard-Oxford atlas and its 25% threshold being parameters. The emotion task was chosen here because its two opposite phase-encoding runs are the minimum structure that makes both the ICC and the run covariate identifiable.

#### Runs Per Subject

The specification generalises directly to more than two runs, and favourably: ICC precision improves from SD *= 0.081* with two runs to 0.026 with ten, and with three or more the random-slope model becomes viable (Sect. “Simulation Analyses”). Subjects contributing a single run are retained and inform the fixed effects, but not the within-subject variance component.

#### External Datasets

Two dataset-specific conventions, neither of them statistical, are the most likely points of failure outside HCP: the file layout of the motion regressors, and the unit convention for rotational parameters (HCP stores degrees, fMRIPrep radians). The pipeline searches candidate paths and takes the rotation unit as an explicit argument, raising errors rather than proceeding with degraded inputs.

#### Surface and CIFTI Data

The implementation operates on volumetric NIfTI. Extending it to CIFTI requires replacing only the masking and map-writing layer, since the statistical core takes an observations-by-vertices matrix and is indifferent to spatial representation.

#### Computational Scaling

Cost is linear in voxels and approximately linear in subjects over the range tested (Fig. 5), so the figures in Sect. “Computational Efficiency” extrapolate reasonably; memory is dominated by the observations-by-voxels matrix and controlled by chunk size.

### Limitations

Key limitations include: (1) single contrast and dataset (HCP emotion task, 142 healthy adults)—the pipeline is designed for generality (Sect. “Generalizability and Extensibility”) but has been validated empirically only here; (2) two runs per subject limit ICC precision and preclude random-slope models, and in the lowest-reliability regions the between-subject variance estimate reaches its lower boundary; (3) random-intercept-only specification; (4) univariate (voxel-by-voxel) analysis; (5) reliance on ICA-FIX without explicit physiological noise modeling; (6) spatial correlation may partially violate FDR independence assumptions; (7) stability analyses were ROI-level only; and (8) volumetric rather than surface-based analysis.

A further limitation is inherited from the acquisition protocol rather than from our design. The HCP acquires the LR run before the RL run for every subject, without counterbalancing. The “run effect” we report is therefore confounded with acquisition order, and may partly reflect practice, adaptation, or fatigue in addition to phase-encoding direction. Our data cannot separate these contributions; doing so would require a counterbalanced acquisition. This does not affect the decomposition result, the run covariate absorbs systematic run-level variance whatever its origin, but it does qualify the mechanistic interpretation, and the term is better read as “run/order effect”.

## Conclusions

We have presented a validated, benchmarked, containerised pipeline for linear mixed-effects analysis of task fMRI. The implementation agrees with the R reference standard to the sixth decimal place, recovers known parameters across five variance regimes, converges on 100% of voxels, and completes a whole-brain analysis of 215,265 voxels in under six hours on twelve cores at *19× * the per-voxel cost of the GLM. It declares its fitting backend explicitly, versions its outputs, and records the environment that produced every result.

Applied to the HCP emotion task, the pipeline shows that GLM and LME produce highly concordant activation patterns, and that the sensitivity difference between them is attributable to the run covariate rather than to hierarchical modeling: an intermediate model retaining the hierarchy but omitting the run term is almost indistinguishable from the GLM. LME provides additional information that summary statistics discard—spatially heterogeneous ICC maps and run effects that survive motion adjustment at two levels, at a computational cost that is manageable for whole-brain analysis. Rather than replacing the GLM, LME is a complement, most valuable when multiple runs are available and within-subject variability is of interest.

The decomposition strategy the pipeline implements, fitting full, intermediate, and standard models over an identical voxel set, generalises beyond run effects to any run-level covariate, and we offer it as a reusable diagnostic for attributing sensitivity differences between statistical approaches in neuroimaging.

## Information Sharing Statement

The HCP data analysed in this study are publicly available from the Human Connectome Project (WU-Minn Consortium) at https://db.humanconnectome.org, subject to the HCP data use terms. All analysis code, including the Dockerfile, pinned environment manifests, and scripts to reproduce every figure and table, is openly available at https://github.com/DaniOrze/hcp-emotion-lme under the MIT license.

## Supplementary Information

Below is the link to the electronic supplementary material.

Supplementary file 1 (pdf 238 KB)

## Data Availability

Data used in this study were provided by the Human Connectome Project, WU-Minn Consortium (Principal Investigators: David Van Essen and Kamil Ugurbil; 1U54MH091657) and are available at https://db.humanconnectome.org.

## Code Availability

Analysis code is available at https://github.com/DaniOrze/hcp-emotion-lme.
