# Supplementary material for: Beyond the General Linear Model: Linear Mixed-Effects Modeling of fMRI Data from the Human Connectome Project
Source: Neuroinformatics. 2026 Sep 29;24(4):64. doi: 10.1007/s12021-026-09820-2 (PMC13624029; doi:10.1007/s12021-026-09820-2)
Supplement: Supplementary file 1 [file 12021_2026_9820_MOESM1_ESM.pdf]

Supplementary Material

Beyond the General Linear Model: Linear Mixed-Effects Modeling of fMRI Data from the Human Connectome Project

Daniele Orzechowski and Ronaldo Martins da Costa

Supplementary Table S1

Conventional between-subject Cohen's d for the GLM, alongside the total-variance d reported in Table 6 of the main text. The two coefficients answer different questions and are not interchangeable.

Between-subject d divides the mean subject-level beta by its standard deviation across subjects ($n = 142$), and therefore describes the effect relative to individual differences. Total-variance d divides by $\sqrt{\mathrm{Var}(\beta_{ij})}$ computed over all 283 run-level observations, which additionally includes within-subject variance and consequently yields smaller values. We report the latter in the main text because it places the GLM and the LME on a common scale, the LME estimate being defined with respect to both variance components.

**ROI**	$\bar{\beta}$ (SD)	d_{between}	d_{total}
L Amygdala	0.237 (0.141)	1.682	1.382
R Amygdala	0.239 (0.124)	1.933	1.476
L Hippocampus	0.045 (0.089)	0.510	0.397
R Hippocampus	0.062 (0.086)	0.722	0.580
Insular Ctx	−0.015 (0.046)	−0.317	−0.245
IFG pars oper.	0.071 (0.082)	0.867	0.630
IFG pars triang.	0.104 (0.090)	1.153	0.935
Anterior Cing.	−0.008 (0.054)	−0.146	−0.107
Paracingulate G.	0.146 (0.115)	1.271	1.040
Medial Frontal Ctx	0.057 (0.085)	0.671	0.545
Sup. Frontal G.	0.007 (0.043)	0.167	0.123
Mid. Frontal G.	0.076 (0.068)	1.116	0.820
Frontal Pole	0.014 (0.025)	0.541	0.377
Orbital Frontal Ctx	−0.014 (0.085)	−0.162	−0.123
L Thalamus	0.048 (0.068)	0.716	0.532
R Thalamus	0.021 (0.060)	0.340	0.274
L Caudate	0.033 (0.107)	0.309	0.220
L Putamen	0.049 (0.085)	0.572	0.423
L Pallidum	−0.004 (0.115)	−0.037	−0.028
Brainstem	0.013 (0.035)	0.362	0.283
L Cerebral Ctx	0.021 (0.021)	1.038	0.832
**Mean $\lvert d \rvert$**		**0.697**	**0.542**

$\bar{\beta}$, mean subject-level beta (BOLD units); SD, standard deviation across subjects; L, left; R, right; Ctx, cortex; G., gyrus; Cing., cingulate; IFG, inferior frontal gyrus; oper., opercularis; triang., triangularis; Sup., superior; Mid., middle. As a consistency check, the between-subject coefficient satisfies $d_{\text{between}} = t/\sqrt{n}$ to four decimal places for all 21 regions, where t is the one-sample statistic reported in Table 6.
